# Beakiation: how a novel parrot gait expands the locomotor repertoire of living birds

**DOI:** 10.1098/rsos.231397

**Published:** 2024-01-31

**Authors:** Edwin Dickinson, Melody W. Young, Michael C. Granatosky

**Affiliations:** ^1^ Department of Anatomy, New York Institute of Technology College of Osteopathic Medicine, Old Westbury, New York, NY, USA; ^2^ Center for Biomedical Innovation, New York Institute of Technology College of Osteopathic Medicine, Old Westbury, New York, NY, USA

**Keywords:** exaptation, innovation, suspensory locomotion, biomechanics, brachiation, *Agapornis roseicollis*

## Abstract

Occupation of arboreal habitats poses myriad locomotor challenges, driving both anatomical and behavioural innovations across various tetrapod lineages. Here, we report and biomechanically assess a novel, beak-driven locomotor mode—‘beakiation’—by which parrots advance along the underside of narrow arboreal substrates. Using high-speed videography and kinetic analyses, we describe the limb loading patterns and pendular mechanics of beakiation, and compare the biomechanical characteristics of this gait with other suspensory behaviours (namely, forelimb-driven brachiation and inverted quadrupedal walking). We report that the parrot beak experiences comparable force magnitudes (approx. 150% body weight in the normal plane; approx. 50% body weight in the fore–aft plane) to the forelimbs of brachiating primates. Parrot beakiation is also characterized by longer-than-expected pendular periods, similar to observations of gibbon brachiation. However, in terms of mechanical energy recovery, beakiation is typified by lower levels of energetic recovery than brachiating specialists: a product of its slower, more careful nature. The observation of this novel behaviour—which adds to a growing base of literature regarding beak-assisted locomotor strategies in birds—highlights the extraordinary behavioural plasticity of birds, the functional versatility of the avian beak, and the difficulties in reconstructing an animal's locomotor repertoire from morphological characteristics alone.

## 1. Introduction

A central tenet of functional morphology—particularly within the context of palaeontological reconstructions of extinct behaviours—is that specific morphological traits or configurations are indicative of an animal's behavioural repertoire [1]. Prominent examples of this form–function relationship include the reconstruction of diet from dental micromorphology [2–4], mating strategies from patterns of body size dimorphism [5–10] or communication abilities from the presence of sound-producing organs [11–13]. Within the context of locomotion, several traits—including gross limb proportions, articular morphologies of the pelvic and pectoral girdles, and various aspects of digital morphology—have been directly or indirectly associated with locomotor speed [14–16], substrate usage [17–21], or gait patterns [22–25].

While such relationships have been robustly explored across numerous lineages, one obvious shortcoming is that behavioural reconstructions using anatomical traits alone can—at best—provide a *minimum* estimate of the range of behaviours used by a particular species, as opposed to a full behavioural repertoire. Moreover, behavioural interpretations drawn from morphology are easily obfuscated by: (i) the development of novel behaviours within a single group among a broader lineage, (ii) the loss of ancestral behaviours by one or more members of a crown group, and (iii) convergence upon shared behaviours by morphologically disparate taxa. Thus, the imperfect match between anatomy and behaviour is inherently conservative and can overlook the specific novel innovations that most interest evolutionary biologists.

### 1.1. Locomotor innovations within an arboreal context

Occupation of the arboreal milieu imposes many locomotor demands upon animals, who must navigate a complex three-dimensional environment defined by discontinuous structures that vary in size, orientation and compliance. To successfully navigate this challenging landscape, arboreal animals exhibit high locomotor diversity [26], making use of a range of innovative gaits and locomotor strategies that include tail-suspension [27–29], lasso climbing [30,31], ricochet brachiation [32–35], mitten gliding [36–39] and beak-assisted vertical climbing [40–42]. While multiple avian groups (including vultures and tropicbirds) will use their beaks to assist in terrestrial locomotion [43,44], beak-assisted climbing—in which birds load their beak as a third limb to support their body weight when scaling vertical surfaces—was recently described in numerous parrot taxa across a range of body sizes in an arboreal context [40–42,45,46]. Additionally, a single account [47] describes use of the beak as a suspensory apparatus during horizontal arboreal movement in a single passerine taxon (*Spindalis portoricensis*; the Puerto Rican spindalis). Thus, the avian beak represents a diverse locomotor organ capable of assisting in multiple forms of both terrestrial and arboreal movements.

In this study, we describe the first usage of suspensory beak-driven locomotion—hereafter ‘beakiation’—within a model parrot species (*Agapornis rosiecollis;* the rosy-faced lovebird) and provide the first quantitative analysis of this gait in any taxon: reporting movement speeds and gait patterns across several trials. We also collect data on the limb loading and pendular mechanics associated with this behaviour and compare the efficiency of this mechanism against other suspensory behaviours (e.g. inverted quadrupedal walking in suspensory taxa, brachiation in gibbons and humans). In so doing, we provide a quantitative and comparative perspective on this novel locomotor behaviour.

## 2. Material and methods

### 2.1. Study sample

Experimental data were collected from four rosy-faced lovebirds (*Agapornis roseicollis*) housed at the New York Institute of Technology College of Osteopathic Medicine (Old Westbury, NY, USA). All work followed protocols approved by New York Institute of Technology College of Osteopathic Medicine Institutional Animal Care and Use Committee (IACUC protocol: 2021-MG-03). All animal subjects were adults (0.050 ± 0.002 kg in weight) in good health and presented free of any visible pathologies or gait abnormalities. Animals retained the ability to fly short distances, and indeed typically returned independently to their housing apparatus after each trial in this manner.

### 2.2. Experimental design

**Figure 1.**
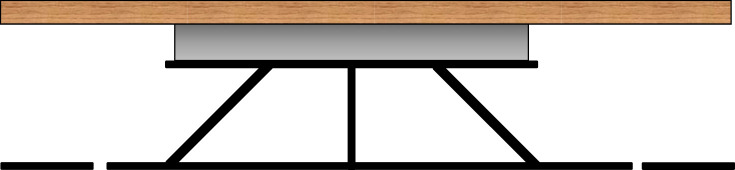
Schematic illustration of experimental set-up, showing attachment of the suspended runway (black) to the force plate (silver). Note that the uninstrumented portions of the runway (far left and far right, respectively) extend beyond frame (for exact dimensions of runway see Material and methods).

Two distinct experimental designs were employed to analyse the centre of mass (COM) movements and single limb forces associated with beakiation, respectively. To collect COM data, animals were presented with a simulated arboreal support (67.5 cm in total length, 2.5 mm in diameter) three-dimensional printed in Onyx material using an Markforged Mark Two printer (Waltham, MA, USA) in three components (each 22.5 cm in length and 2.5 mm in diameter). The middle was instrumented with a small-load force plate (model HE6X6; Advanced Mechanical Technology, Inc., Watertown, MA, USA) attached upside down to a large, flat wooden platform. The remainder of the runway was mounted flush to the wooden platform with a small gap on either end to prevent runway interference (figure 1 and electronic supplementary material, video S1). The runway was suspended 45 mm under the wooden platform. In total, 129 successful trials were conducted using this set-up.

Single limb forces were collected using a modified version of this apparatus following [48–51], in which the length of the support was reduced to 49.5 cm (comprising two uninstrumented 22.5 cm sections flanking a single instrumented 4.5 cm section). A smaller instrumented section was used for this second experiment to isolate the contact of a single hindlimb or beak without interference from other points of contact. The diameter of the support remained at 2.5 mm and was again three-dimensional printed in Onyx material using the same device and instrumented via the same force plate. In total, 500 strides from 142 distinct locomotor trials were conducted using this set-up.

### 2.3. Data collection

All trials began by taring the force plate to remove any drift or offsets from previous recordings. The animals had been previously acclimatized to the experimental set-up and were comfortable moving along the length of each runway. No explicit training was used; rather, animals were placed upon the runway and moved voluntarily across it in a manner of their choosing. Thus, this should not be considered an ‘optimized’ behaviour but rather facultative given the specific set-up faced by the animals, and interpretations should be drawn thusly. Movements of the bird along the runway were recorded using two high-speed cameras (XC-2; Xcitex Inc., Woburn, MA, USA) positioned to capture lateral and posterior views at 125 Hz. These cameras were synchronized to our force plate (collecting at 1250 Hz) using the software Procapture (Xcitex Inc., Woburn, MA, USA).

### 2.4. Data processing

Spatio-temporal variables (i.e. contact time, total stride time, duty factor, stride length, stride frequency and velocity) were measured from lateral video footage of locomotor trials in ImageJ [52]. A known distance in the background of the video was used to calibrate the space. Touchdown, lift-off and subsequent touchdown frames of each stride were recorded and divided by the recording frame rate to obtain total stride time. Contact time was calculated by taking the difference between initial touchdown and lift-off. Duty factor was calculated by dividing contact time by total stride time. Stride length was calculated by multiplying speed by stride time. Stride frequency is the inverse of stride time. Speed was calculated by tracking each individual's COM between each frame within a stride and determining the amount of time required to traverse a known distance on the runway. Expected pendular period (*T*) was calculated as

$$T=2\pi \;\surd \;\frac{L}{g},$$

where *T* is pendular period, *L* is the distance between the substrate contacting beak and the animal's COM, and *g* is gravitational acceleration (9.81 m s^−2^) [25]. This original COM position was placed manually in ImageJ using the authors' own data on COM position in rosy-faced lovebirds, which was collected using the double balance-board method (following [53]) and has been previously published [42]. The expected pendular period was calculated using the same variables described above (see introduction). Stride time was used as the observed pendular period.

To analyse COM movements, only trials which contained a full stride (i.e. leading limb touchdown to subsequent touchdown of the same limb) were kept and analysed through custom-written Matlab (Natick, MA) code. Briefly, the whole-body ground reaction forces registered from the force plate were divided by body weight to calculate acceleration. These accelerations were then integrated for instantaneous velocity and subsequently instantaneous position in each cardinal plane (i.e. fore–aft, normal and mediolateral). To derive energetic costs, we followed previously published methodology [54–59] wherein kinetic energy (EK) in the fore–aft (EKx), tangential (EKz) and mediolateral (EKy) planes were calculated as

$$EK=\;\frac{1}{2}mv^{2},$$

where *m* is the mass of the animal and *v* is the velocity of the COM in each respective axis. Potential energy of the COM (EP) was calculated using

EP= mgh,

where *m* is the mass of the animal and *g* is gravitational force (9.81 m s^−2^) and *h* is the vertical displacement in each plane COM in each respective axis. Total fore–aft, mediolateral and tangential kinetic (EK) and potential energies (EP) were summed during each stride to obtain total energy (ET) of the COM. Pendular effectiveness was estimated as per cent recovery following [60]

$$\mathrm{per}\;\mathrm{cent}\;\mathrm{recovery}\;(\text{\%})=\;\frac{(\Delta EK+\Delta EP)-\Delta ET}{\Delta EK+\Delta EP}\times 100$$

where ΔEK, ΔEP and ΔET are the sum of the positive increments of the EK, EP and ET profiles, respectively.

To analyse single limb forces, only trials in which there was a clear separation between beak and hindlimbs contacts were analysed and run through a custom-written Matlab script to reflect direction of travel, orientation and whether the contacting limb was right or left [59]. The fore–aft and normal forces were standardized to reflect those forces applied to the animal by the force plate, such that fore–aft forces were defined as a positive propulsive and negative braking force, normal forces towards the plate reflected positive values, and those pulling away from the plate were negative. For presentation purposes, however, as the locomotor mode was exclusively below-branch, all normal forces are displayed as positive values reflecting the magnitude of pulling force. All forces were filtered through a low-pass Fourier filter at 15 Hz [61], and peak fore–aft and normal forces were extracted from the beak and hindlimbs single limb footfalls. All peak force data were normalized using the animal's body weight (% BW) to allow statistical comparison between individuals (see below) and comparison with other below-branch taxa derived from the literature.

### 2.5. Statistical analyses

Differences between expected and observed pendular periods were assessed via paired *t*-tests. To understand differences in limb loading patterns between the beaks and hindlimb, we constructed three linear mixed-effect models (one each for the propulsive, normal and mediolateral planes). Data were checked for normality and heteroskedasticity and rank transformed to satisfy the assumptions of all statistical tests. Each model held limb (beak or hindlimb) and velocity as fixed effects and included individual as a random effect (to account for individual idiosyncrasies). All analyses were conducted in R [62] and are reported as a table in electronic supplementary material, table S1.

## 3. Results

**Figure 2.**
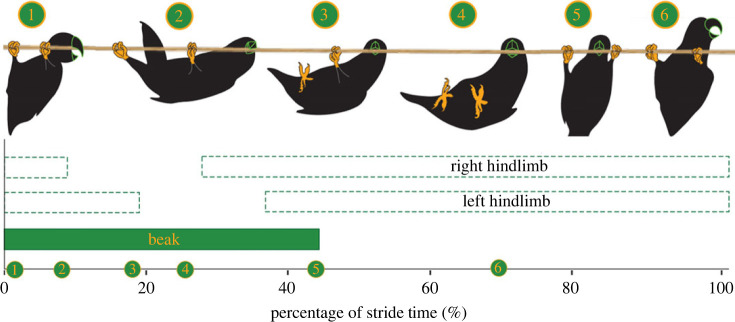
Representative gait diagram of beakiation within parrots, accompanied by silhouettes detailing the novel gait pattern. For display purposes, the strides begin and end with a beak touchdown. Beakiation involves an alternating gait pattern in which: (i) the beak grasps the support; (ii) the two hindlimbs release synchronously, swinging the centre of mass forward while pivoting about the beak; (iii) the hindlimbs re-engage at an advanced point along the substrate; and (iv) the beak assumes a new grasping position in front of the hindlimbs.

Beakiation was observed as an alternating gait pattern in which the beak first grasps the support, before both hindlimbs release near synchronously such that the bird pivots about its beak to swing its COM forward. The hindlimbs then re-engage the substrate at a new position, and the beak assumes a new grasping position in front of the hindlimbs (see electronic supplementary material, movie S1 and figure 2). The average stride velocity was approximately 0.10 m s^−1^ with a stride length of 0.07 ± 0.01 m and an average stride frequency of 1.33 ± 0.27 Hz. Average contact time was 0.46 ± 0.16 s (duty factor = approximately 57.70%) for the beak, and 0.61 ± 0.23 s (duty factor = approximately 77.60%) for the hindlimb.

**Figure 3.**
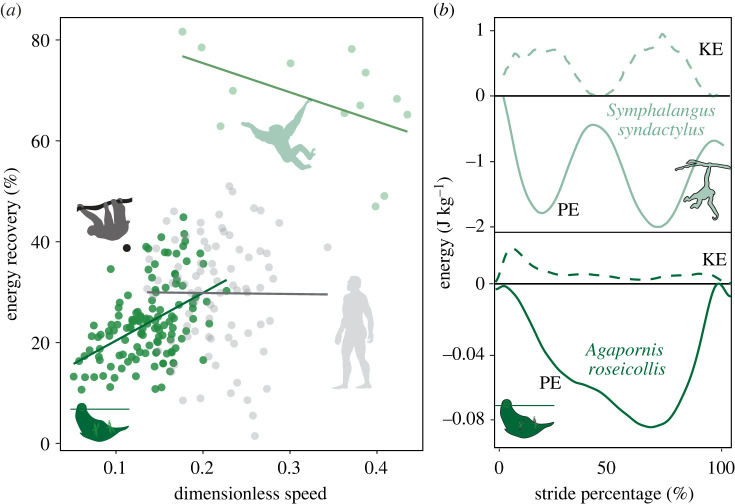
(*a*) Comparative pendular mechanics of below-branch locomotion, showing energy recovery (%) during beakiation in parrots (*Agapornis roseicollis*) compared with brachiation in non-human primates (*Hylobates moloch, Ateles fusciceps, Pygathrix nemaeus*), brachiation in humans (*Homo sapiens*) and inverted quadrupedal walking in three-toed sloths (*Bradypus variegatus*) plotted against dimensionless speed. (*b*) Representative energy traces of beakiation in a parrot (*Agapornis roseicollis*) compared with brachiation in a gibbon (*Symphalangus syndactylus*). Kinetic energy depicted with dashed lines, potential energy depicted with solid lines. All comparative data were drawn from literature values [35,48–51,63–66].

**Figure 4.**
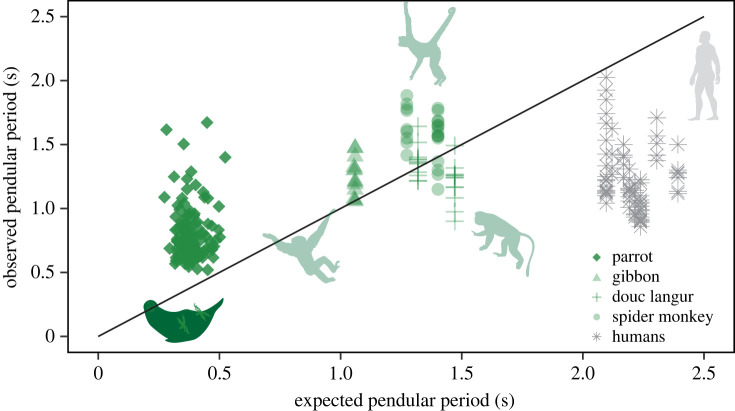
Bivariate plot of observed pendular period against expected pendular period in parrots (*Agapornis roseicollis*), brachiating non-human primates (*Hylobates moloch, Ateles fusciceps, Pygathrix nemaeus*) and brachiating humans (*Homo sapiens*). Black line indicates a perfect pendular system. All comparative data were drawn from literature values [64,65].

**Figure 5.**
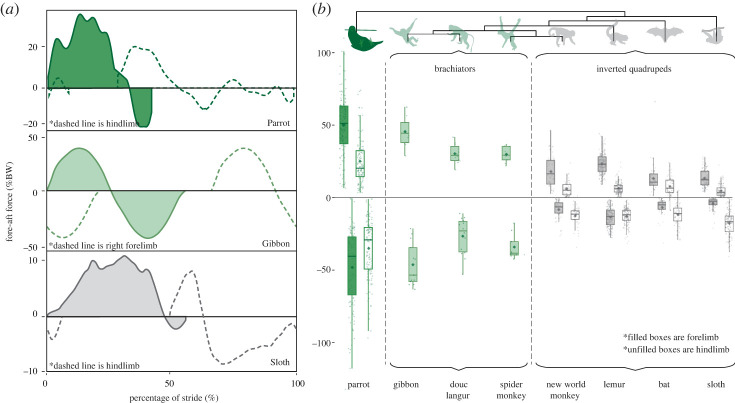
(*a*) Representative fore–aft plane force traces of a parrot (*Agapornis roseicollis*) compared with a representative brachiator (gibbon; *Hylobates moloch* [64]) and a representative inverted quadrupedal walker (sloth; *Bradypus vareigatus* [50,51]). The filled solid line represents the beak (parrot), forelimb (sloth) or left forelimb (gibbon); unfilled dashed line represents the hindlimb (parrot and sloth) or right forelimb (gibbon). All forces normalized to body weight (% BW). Positive values indicate propulsive forces, negative values indicate braking forces. (*b*) Box-and-whisker plots of fore–aft forces during beakiation in parrots compared with brachiating non-human primates (*Hylobates moloch, Ateles fusciceps, Pygathrix nemaeus*; light green) and walking in inverted quadrupeds (grey; new world monkey is a grouped average of *Saimiri sciureus*, *Aotus nancymae* and *Cebus capucinus*; lemur is a grouped average of *Daubentonia madagascariensis*, *Lemur catta*, *Propithecus coquereli* and *Varecia variegata;* bat is a grouped average of *Pteropus vampyrus* and *Desmodus rotundus;* sloth is a grouped average of *Bradypus variegatus* and *Choloepus didactylus*). Filled bars represent forelimb forces and unfilled bars represent hindlimb forces. As in (*a*), all forces normalized to body weight (% BW); positive values indicate propulsive forces, negative values indicate braking forces. All comparative data were drawn from literature values [35,48–51,63–66].

**Figure 6.**
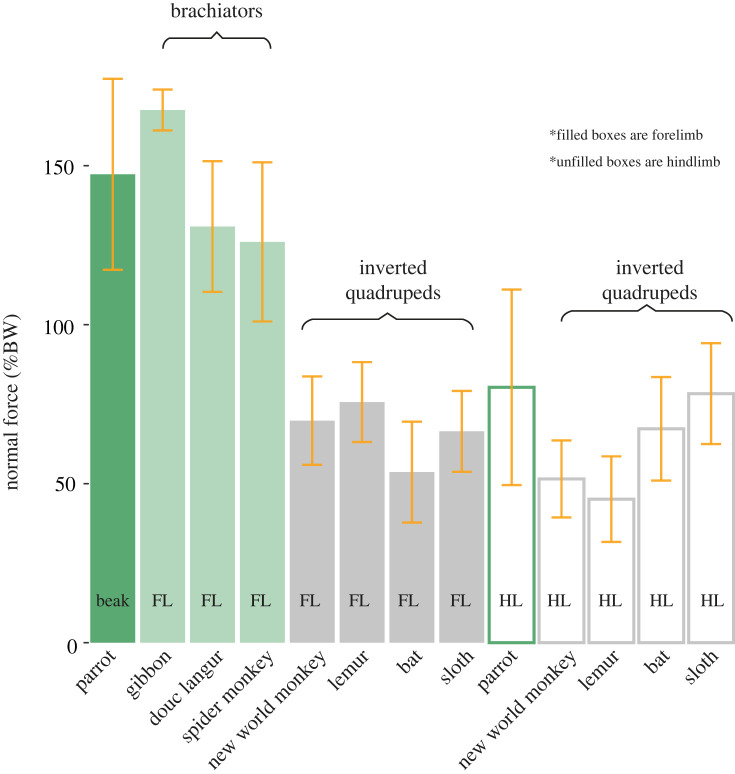
Comparison of single limb forces in the normal plane, normalized to body weight (% BW). Data from parrots (*Agapornis roseicollis*) are compared with brachiating primates (*Hylobates moloch, Ateles fusciceps, Pygathrix nemaeus*; light green), and inverted quadrupeds during walking (grey; new world monkey is a grouped average of *Saimiri sciureus*, *Aotus nancymae* and *Cebus capucinus*; lemur is a grouped average of *Daubentonia madagascariensis*, *Lemur catta*, *Propithecus coquereli* and *Varecia variegata;* bat is a grouped average of *Pteropus vampyrus* and *Desmodus rotundus;* sloth is a grouped average of *Bradypus variegatus* and *Choloepus didactylus*). Filled bars represent beak or forelimb forces, unfilled bars represent hindlimb forces. All comparative data were drawn from literature values [35,48–51,63–66].

From a total of 129 strides collected from our COM experimental runway, we observed that parrots recovered an average of 23.83 ± 7.47% of energy per stride (figure 3). We additionally observed longer than expected pendular periods (observed = 0.80 ± 0.21 s, expected = 0.38 ± 0.05 s, *p* < 0.001; figure 4). From a total of 250 single limb forces (125 beaks and 125 hindlimbs), we observed that the parrot beak (50.22 ± 20.81% BW, figure 5) imparted double the propulsive force magnitude (*p* < 0.001) of the hindlimb (25.1 ± 17.9% BW), and greater subsequent braking forces (beak = −48.00 ± 25.75% BW, hindlimb = −34.79 ± 20.41% BW, *p* < 0.001; figure 5). Additionally, in the normal plane, the parrot beak experiences nearly double the magnitude (*p* < 0.001) that of the hindlimbs (beak = 147.30 ± 30.04% BW; hindlimb = 80.29 ± 30.75% BW; figure 6). Velocity did not significantly covary with force in any models (all *p*-values > 0.140).

## 4. Discussion

Here, we present the first biomechanical analysis of below-branch locomotion within parrots using a model species (*Agapornis roseicollis)*, and contextualize the mechanics of this gait against other specialized suspensory tetrapods [48,50,51,63,67]. We demonstrate that parrots employ a distinct form of locomotion we coin beakiation, in which the beak initially secures a grip on the support followed by the simultaneous release of both hindlimbs. This action allows the bird to pivot around its beak and propel its COM forward. The hindlimbs then contact the substrate further along its path, while the beak assumes a new grasping position ahead of the hindlimbs (figure 2). Beakiation represents a novel and previously undocumented movement pattern that expands the range of locomotor behaviours observed in birds. It is also behaviourally differentiated from the beak-suspension previously reported in the Puerto Rican spindalis [47], insofar as beakiation involves coordinated movements of the beak and hindlimbs, as opposed to being a purely beak-driven gait.

It should be emphasized here that this analysis was conducted in an experimental context (i.e. these were animals performing this behaviour in a laboratory where the videographic and analytical apparatus could be established), as opposed to in their natural habitat. As discussed above, beak-suspension is not reported in any wild parrot (nor any wild bird beyond one single, oral account in the Puerto Rican spindalis that lacks either photographic or videographic evidence). Accordingly, we encourage caution when interpreting these data and do not assert that this behaviour is necessarily adaptive. Nevertheless, we consider this an important observation that requires formal biomechanical analysis of this nature. First, this example neatly underscores the behavioural diversity of which the parrot beak (and broader cranio-cervical apparatus) is capable, and is particularly impressive given the large loading regime experienced by the feeding system during this behaviour. Moreover, parrots have extensive documented histories of both beak-driven locomotion in other contexts [40,41,68] and of adopting suspensory behaviours in other contexts (e.g. hanging parrots, who are unique among avians in choosing to sleep while suspended upside-down in a similar manner to roosting bats [69]). Thus, we consider it entirely plausible—perhaps likely—that this behaviour may be used in specific contexts in the wild, much like beak-swinging in spindalids. Indeed, the absence of behavioural reports speaks to a larger issue in both ornithology and broader studies of animal behaviour: namely, the lack of quantitative observational studies based on common ethograms. While such reports are abundant in specific lineages—most notably primates, who boast hundreds of behavioural reports that list minutiae behaviours over large observational spans [26]—there is to date only a single quantitative, ethogram-driven study of avian behaviours. This study, which includes more than 11 000 observations of a colony of monk parakeets in Brooklyn, New York, incidentally noted that suspensory behaviours broadly constituted 0.36% of the locomotor repertoire [70] and serves as a valuable example for future, focused studies of the behavioural repertoire of other parrot species.

Unlike traditional brachiation observed in primates [35,64,65,71], beakiation involves a 90° lateral reorientation of the body axis (electronic supplementary material, video S1). This sideways reorientation probably reflects anatomical constraints associated with the avian skeleton [72]. Birds have extensively modified their pelvic girdle and lower limb relative to most other living tetrapods: consolidating multiple tarsal and metatarsal bones into a single tarsometatarsus, and exhibiting a pelvic antitrochanter and tight ligaments in the hip that strongly limit femoral abduction (instead relying upon long-axis rotation of the femur and tibiotarsus to control position of the pes; [72,73]). In part due to this morphotype, birds similarly adopt a lateral body position when bipedally traversing narrow substrates: a behaviour known as ‘sidling’, in which forwards progression along a branch occurs via a side-to-side shuffling gait in which the ventral surface of the body is orthogonal to the axis of motion [74]. In this context, it is perhaps unsurprising that beakiation is also characterized by a similar sideways reorientation. Additionally, owing to modifications of the avian forelimb for flight, beakiation necessarily differs from brachiation by using the head, in place of the forelimb, as the leading point of contact. Exaptation of the parrot head into the locomotor cycle is not unique to beakiation; rather, the head and neck of parrots are similarly used as a third limb (and experience similar loading magnitudes to the hindlimbs) when scaling vertical surfaces [41,45–47,68]. However, the general gait pattern observed during beakiation is distinct from beak-assisted climbing insofar as the hindlimbs are moving together in near synchrony. Beakiation, therefore, can be functionally considered as a two-point-of-contact gait involving cyclical interchange between the craniocervical system and the paired hindlimbs—a movement pattern that closely resembles two-link brachiating robots [75,76]. Thus, the parrot craniocervical apparatus represents a highly flexible neuromuscular system capable of solving numerous locomotor challenges [45]. Indeed, in light of these collective recent findings, we suggest that the avian cranio-cervical system may be considered a fourth locomotor module (*sensu* [77])—functioning independent of the hindlimb, wing and tail to facilitate new behaviours (e.g. vertical climbing, suspensory walking) in an arboreal environment.

### 4.1. Kinetics of beakiation

The novelty of this behaviour lies in the observed functional convergence between the parrot beak during beakiation and the forelimbs of brachiating primates. During the unsupported phase of beakiation in parrots (i.e. when only the beak is in contact with the substrate), the head assumes a crucial role, generating normal forces nearly 150% of the bird's body weight (figure 6). In so doing, parrots are loading their heads at peak magnitudes matching, if not exceeding, the loading environments of the forelimb in specialized brachiators like gibbons (approx. 170% BW) [33,64], douc langurs (approx. 130% BW) [64,66], and spider monkeys (125% BW) [64]. Similarly, in the fore–aft plane, parrots generate propulsive and braking forces that exceed 50% BW (figure 5), greater than the equivalent forces seen in the forelimbs of both brachiating primates and non-specialized inverted quadrupeds (figure 5; [48,50,51,63,64,67]). Thus, beakiation represents a true beak-driven mode of locomotion, in which the craniocervical system assumes a critical role in both supporting the body and generating propulsive movements.

The force-generating capacities of the parrot beak are well known, with *in vivo* forces registering at 37 times their body weight [78]. Similarly, the neck muscles of parrots are exceptionally strong, with neck flexors capable of generating forces up to 28 times body weight (see electronic supplementary material, table S2). While such a force generating capacity is not essential to power beakiation (as evidenced by the magnitudes of forces generated by our parrots during locomotor trials), a strong neck may provide a valuable safety factor that facilitates the ease of this behaviour to parrots. Limited data on neck strength exists in other taxa, though comparisons with humans (neck flexion force = 19% BW; neck extension force = 34% BW; [79]) and domestic dogs (for anatomical estimates of neck muscle strength, see [80]) would suggest that the parrot neck is exceptionally strong compared with these taxa. However, comparative data on the strength of the head and neck in other avian taxa—particularly other taxa capable of beak-suspension, such as spindalids [47]—are necessary to empirically evaluate the extent to which a powerful neck is a prerequisite for this locomotor mode.

### 4.2. Pendular mechanisms during beakiation

Given the stark similarity in limb loading patterns between beakiation and brachiation, it might be assumed that parrots also adopt similar biomechanical mechanisms to gibbons and other dedicated brachiating primates. As brachiators swing beneath branches, they effectively achieve an oscillatory motion resembling those of a pendulum [32,35,65,81]. Perfect pendular systems (i.e. existing in a space devoid of air resistance or friction) are capable of transforming potential into kinetic energy so that the absolute maximum magnitudes of each energetic component are reached simultaneously (e.g. potential energy reaches its absolute minima as kinetic energy reaches its absolute maxima) [82]. Gibbons capitalize on these mechanics, such that they achieve a near seamless exchange of potential and kinetic energy and recover nearly 80% of the initial energetic input from stride to stride [32,35,65,81]. Contrary to our predictions, however, parrots recover relatively little mechanical energy per stride (figure 3), at a similar level to non-specialized brachiators like humans (approx. 25%) [65]. There are probably a few mechanistic explanations for this discrepancy [83]. First, beakiation occurs at significantly lower speeds than brachiation (dimensionless speeds of 0.14 versus 0.33 for beakiation and brachiation, respectively). As such, less kinetic energy is accumulated throughout the stride. Secondly, beakiation is start–stop in nature: limiting the potential for pendular mechanical energy recovery between strides. Instead, parrots actively decelerate their COM toward the end of a stride, requiring additional muscular effort to regain their lost momentum. Though this strategy is both mechanically and metabolically expensive, it may permit more controlled and careful locomotor movements, a benefit which may be critical to successfully navigating complex and discontinuous arboreal environments [83].

Pendular period, or the time necessary to complete a full cycle (e.g. forward and back swing in a true pendulum, and touchdown to touchdown of the same point of contact in an animal) is another mechanical parameter that may be compared between locomotor modes [64,65]. Calculated from the length of the pendulum *L* (i.e. the distance between the pivot point and COM of object/animal, see equation above), simple gravity pendulums achieve equidistant and isochronous swings. Parrots, like gibbons, have longer pendular periods than would be expected based on their *L (*i.e. distance from beak point of contact to COM), a trait probably attributable to active behavioural modulation during locomotion [64,65]. Brachiation is associated with relatively high injury rates compared with other locomotor modes [84], and while it is remains unclear if comparable levels of injury exist for parrots during beakiation, the possible risk of injury serves as rational explanation for why parrots and non-human primates may prioritize adaptability over efficiency when navigating challenging arboreal environments. Thus, the deliberate, slow and steady nature of beakiation provides parrots with more time to assess the structural integrity of a substrate and enact precise limb placement, enhancing overall stability and security while moving across slender arboreal substrates.

### 4.3. Broader implications

The observation of this novel behaviour—which adds to a growing base of literature regarding beak-assisted locomotor strategies in birds [40,41,43,44,47]—underscores the numerous difficulties of using morphology to predict a species' behavioural repertoire. While presumably facilitated by numerous adaptations (e.g. strong jaw and neck musculature, a hook-like beak), these traits are clearly not diagnostic of beakiation: many taxa show enlarged craniocervical muscles to support dietary specializations [78,85–88], while beak shape has been shown to covary with numerous aspects of avian ecology [89–95]. Moreover, as shown by previous anecdotal reports [47] of beak suspension in spindalids—generalized Passeriformes that share few morphological traits with parrots—similar behaviours can be replicated via vastly different morphotypes. Such observations emphasize the multi-dimensional nature of the avian beak as a remarkably fluid structure capable of exaptation towards numerous specialized behaviours [45,46]. Thus, only through detailed ethology [70]—a time-consuming endeavour untranslatable to the fossil record—can biologists hope to document a more complete understanding of behavioural diversity, and from there attempt to unravel the anatomical and physiological correlates of these activities.

### 4.4. Summary

Taken together, beakiation represents a novel mode of suspensory locomotion, primarily driven by the head and neck: which experience similar loading magnitudes to the forelimbs of specialized brachiating primates. Additionally, the longer-than-expected pendular periods observed during beakiation resemble patterns seen in brachiating specialists such as gibbons. However, in terms of mechanical energy recovery, parrot beakiation is characterized by lower rates of energetic recovery than seen in brachiating specialists; instead following similar trends to that observed in non-specialized brachiators like humans.

## Data Availability

Raw data are presented as a electronic supplementary material data file. Additionally, full statistical analysis are presented as electronic supplementary material, tables S1 and S2 [96].
